# *N*-(2-Chloro­benzo­yl)benzene­sulfonamide

**DOI:** 10.1107/S1600536809055482

**Published:** 2010-01-09

**Authors:** B. Thimme Gowda, Sabine Foro, P. A. Suchetan, Hartmut Fuess

**Affiliations:** a Department of Chemistry, Mangalore University, Mangalagangotri 574 199, Mangalore, India; b Institute of Materials Science, Darmstadt University of Technology, Petersenstrasse 23, D-64287 Darmstadt, Germany

## Abstract

The asymmetric unit of the title compound, C_13_H_10_ClNO_3_S, contains two independent mol­ecules, the chloro­phenyl ring of one of them being disordered over two orientations with occupancies of 0.836 (2) and 0.164 (2). In one of the independent mol­ecules, the sulfonyl-bound phenyl ring and the chloro­phenyl ring form dihedral angles of 87.3 (1) and 46.8 (1)°, respectively, with the –S–NH–C=O segment, while in the other mol­ecule the corresponding angles are 76.0 (1) and 39.6 (1)°. In the crystal, mol­ecules are linked into tetra­meric units by N—H⋯O hydrogen bonds.

## Related literature

For background literature and similar structures, see: Gowda *et al.* (2009**a* ▶,b* ▶); Suchetan *et al.* (2009 ▶).
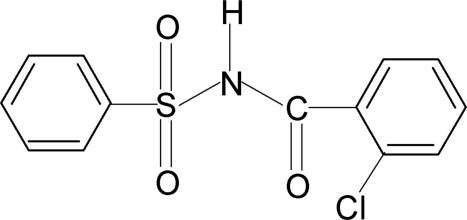


## Experimental

### 

#### Crystal data

C_13_H_10_ClNO_3_S
*M* *_r_* = 295.73
Triclinic, 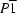

*a* = 7.3390 (5) Å
*b* = 10.828 (1) Å
*c* = 17.685 (1) Å
α = 93.088 (6)°
β = 96.863 (7)°
γ = 103.057 (8)°
*V* = 1354.46 (17) Å^3^
*Z* = 4
Mo *K*α radiation
μ = 0.44 mm^−1^
*T* = 299 K
0.50 × 0.44 × 0.24 mm

#### Data collection

Oxford Diffraction Xcalibur diffractometer with a Sapphire CCD detector
Absorption correction: multi-scan (*CrysAlis RED*; Oxford Diffraction, 2009 ▶) *T*_min_ = 0.811, *T*_max_ = 0.902
9641 measured reflections
5521 independent reflections
4327 reflections with *I* > 2σ(*I*)
*R*_int_ = 0.014

#### Refinement

*R*[*F* ^2^ > 2σ(*F* ^2^)] = 0.041
*wR*(*F* ^2^) = 0.113
*S* = 1.03
5521 reflections
359 parameters
4 restraints
H atoms treated by a mixture of independent and constrained refinement
Δρ_max_ = 0.38 e Å^−3^
Δρ_min_ = −0.34 e Å^−3^

### 

Data collection: *CrysAlis CCD* (Oxford Diffraction, 2009 ▶); cell refinement: *CrysAlis RED* (Oxford Diffraction, 2009 ▶); data reduction: *CrysAlis RED*; program(s) used to solve structure: *SHELXS97* (Sheldrick, 2008 ▶); program(s) used to refine structure: *SHELXL97* (Sheldrick, 2008 ▶); molecular graphics: *PLATON* (Spek, 2009 ▶); software used to prepare material for publication: *SHELXL97*.

## Supplementary Material

Crystal structure: contains datablocks I, global. DOI: 10.1107/S1600536809055482/ci5004sup1.cif

Structure factors: contains datablocks I. DOI: 10.1107/S1600536809055482/ci5004Isup2.hkl

Additional supplementary materials:  crystallographic information; 3D view; checkCIF report

## Figures and Tables

**Table 1:** Hydrogen-bond geometry (Å, °)

*D*—H⋯*A*	*D*—H	H⋯*A*	*D*⋯*A*	*D*—H⋯*A*
N1—H1*N*⋯O1^i^	0.84 (2)	2.09 (2)	2.922 (2)	168 (2)
N2—H2*N*⋯O3^ii^	0.82 (2)	2.06 (2)	2.883 (2)	179 (3)

Symmetry codes: (i) 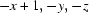; (ii) 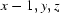.

**Fig. 1.**
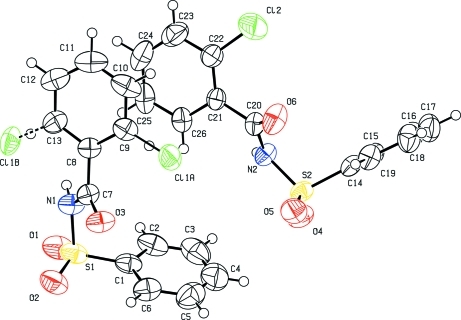
Molecular structure of the title compound, showing the atom-labelling scheme. Displacement ellipsoids are drawn at the 50% probability level. Both disorder components are shown. The minor disorder components are shown with dashed bonds.

**Fig. 2.**
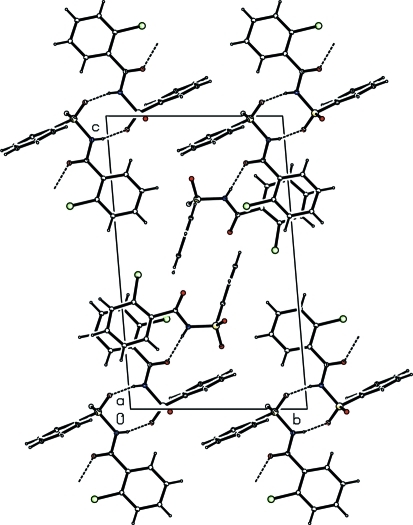
Molecular packing in the title compound. Hydrogen bonds are shown as dashed lines. For clarity, only the major disorder component is shown.

## supplementary crystallographic information

### Comment

Diaryl acylsulfonamides are known as potent antitumor agents against a broad spectrum of human tumor xenografts in nude mice. As part of a study of the effect of ring and the side chain substituents on crystal structures of *N*-aromatic sulfonamides (Gowda *et al.*, 2009*a,b*; Suchetan *et al.*, 2009), in the present work, the structure of *N*-(2-chlorobenzoyl)benzenesulfonamide (I) has been determined (Fig.1).

The asymmetric unit of the title compound contains two independent molecules, A (with S1) and B (with S2). The conformation of the N—H bond in the C—SO_2_—NH—C(O) segment of the structure is *anti* to the C═O bond, similar to that observed in *N*-(benzoyl)benzenesulfonamide (II) (Gowda *et al.*, 2009*a*), *N*-(3-chlorobenzoyl)benzenesulfonamide (III) (Gowda *et al.*, 2009*b*) and *N*-(4-chlorobenzoyl)benzenesulfonamide (IV) (Suchetan *et al.*, 2009).

Both independent molecules are twisted at their *S* atoms; the dihedral angle between the sulfonyl-bound phenyl ring and the S—N(H)—C═O segment is 87.3 (1)° in molecule A and 76.0 (1)° in molecule B, compared to the values of 86.5(0.1) in (II), 89.9 (1)° in (III) and 75.7 (1)° in (IV). Furthermore, the dihedral angle between the chlorophenyl ring and the S—NH—C═O segment is 46.8 (1)° in molecule A and 39.6 (1)° in molecule B.

The dihedral angles between the two phenyl rings in the two molecules of (I) are 69.8 (1)° (molecule A) and 89.8 (1)° (molecule B), compared to the values of 80.3(0.1) in (II), 87.5 (1)° in (III) and 68.6 (1)° in (IV).

The packing of molecules linked by of N—H···O hydrogen bonds (Table 1) is shown in Fig. 2.

### Experimental

The title compound was prepared by refluxing a mixture of 2-chlorobenzoic acid, benzenesulfonamide and phosphorous oxy chloride for 3 h on a water bath. The resultant mixture was cooled and poured into ice cold water. The solid obtained was filtered, washed thoroughly with water and then dissolved in sodium bicarbonate solution. The compound was later reprecipitated by acidifying the filtered solution with dilute HCl. It was filtered, dried and recrystallized. Rod like colourless single crystals of the title compound were obtained by slow evaporation of its toluene solution at room temperature.

### Refinement

One of the Chlorophenyl rings is disordered over two orientations corresponding to a 180° rotation about the C7—C8 bond. The occupancies of the two conformers were refined so that their sum was unity [0.836 (2) and 0.164 (2)]. The C9—Cl1A and C13—CL1B distances were restrained to 1.75 (1) Å.

The H atoms of the NH groups were located in a difference map and refined with the N-H distance restrained to 0.86 (2) %A. The remaining H atoms were positioned with idealized geometry using a riding model with C–H = 0.93 Å. All H atoms were refined with isotropic displacement parameters (set to 1.2 times of the *U*_eq_ of the parent atom).

### Crystal data

**Table:**

C_13_H_10_ClNO_3_S	*Z* = 4
*M**_r_* = 295.73	*F*(000) = 608
Triclinic, *P*1	*D*_x_ = 1.450 Mg m^−^^3^
Hall symbol: -P 1	Mo *K*α radiation, λ = 0.71073 Å
*a* = 7.3390 (5) Å	Cell parameters from 4232 reflections
*b* = 10.828 (1) Å	θ = 2.9–27.8°
*c* = 17.685 (1) Å	µ = 0.44 mm^−^^1^
α = 93.088 (6)°	*T* = 299 K
β = 96.863 (7)°	Rod, colourless
γ = 103.057 (8)°	0.50 × 0.44 × 0.24 mm
*V* = 1354.46 (17) Å^3^	

### Data collection

**Table:**

Oxford Diffraction Xcalibur diffractometer with a Sapphire CCD detector	5521 independent reflections
Radiation source: fine-focus sealed tube	4327 reflections with *I* > 2σ(*I*)
graphite	*R*_int_ = 0.014
Rotation method data acquisition using ω and φ scans	θ_max_ = 26.4°, θ_min_ = 2.9°
Absorption correction: multi-scan (*CrysAlis RED*; Oxford Diffraction, 2009)	*h* = −8→9
*T*_min_ = 0.811, *T*_max_ = 0.902	*k* = −13→13
9641 measured reflections	*l* = −22→21

### Refinement

**Table:**

Refinement on *F*^2^	Primary atom site location: structure-invariant direct methods
Least-squares matrix: full	Secondary atom site location: difference Fourier map
*R*[*F*^2^ > 2σ(*F*^2^)] = 0.041	Hydrogen site location: inferred from neighbouring sites
*wR*(*F*^2^) = 0.113	H atoms treated by a mixture of independent and constrained refinement
*S* = 1.03	*w* = 1/[σ^2^(*F*_o_^2^) + (0.0558*P*)^2^ + 0.4941*P*] where *P* = (*F*_o_^2^ + 2*F*_c_^2^)/3
5521 reflections	(Δ/σ)_max_ = 0.001
359 parameters	Δρ_max_ = 0.38 e Å^−^^3^
4 restraints	Δρ_min_ = −0.34 e Å^−^^3^

### Special details

**Table:**

Geometry. All e.s.d.'s (except the e.s.d. in the dihedral angle between two l.s. planes) are estimated using the full covariance matrix. The cell e.s.d.'s are taken into account individually in the estimation of e.s.d.'s in distances, angles and torsion angles; correlations between e.s.d.'s in cell parameters are only used when they are defined by crystal symmetry. An approximate (isotropic) treatment of cell e.s.d.'s is used for estimating e.s.d.'s involving l.s. planes.
Refinement. Refinement of *F*^2^ against ALL reflections. The weighted *R*-factor *wR* and goodness of fit *S* are based on *F*^2^, conventional *R*-factors *R* are based on *F*, with *F* set to zero for negative *F*^2^. The threshold expression of *F*^2^ > σ(*F*^2^) is used only for calculating *R*-factors(gt) *etc*. and is not relevant to the choice of reflections for refinement. *R*-factors based on *F*^2^ are statistically about twice as large as those based on *F*, and *R*- factors based on ALL data will be even larger.

### Fractional atomic coordinates and isotropic or equivalent isotropic displacement parameters (Å^2^)

**Table:**

	*x*	*y*	*z*	*U*_iso_*/*U*_eq_	Occ. (<1)
Cl1A	0.80617 (12)	0.24676 (7)	0.30758 (5)	0.0701 (3)	0.836 (2)
Cl1B	0.8403 (6)	−0.1454 (3)	0.1258 (2)	0.0654 (14)	0.164 (2)
S1	0.74595 (8)	0.18519 (6)	0.00953 (3)	0.05430 (17)	
O1	0.5985 (3)	0.11237 (18)	−0.04655 (8)	0.0717 (5)	
O2	0.9314 (3)	0.22227 (19)	−0.00917 (10)	0.0716 (5)	
O3	0.9713 (2)	0.22974 (14)	0.16030 (8)	0.0560 (4)	
N1	0.7439 (3)	0.09130 (18)	0.08133 (10)	0.0518 (5)	
H1N	0.649 (3)	0.029 (2)	0.0776 (14)	0.062*	
C1	0.6742 (3)	0.3164 (2)	0.04645 (11)	0.0523 (5)	
C2	0.4851 (4)	0.3057 (3)	0.05382 (14)	0.0684 (7)	
H2	0.3965	0.2303	0.0373	0.082*	
C3	0.4304 (5)	0.4070 (4)	0.08556 (17)	0.0816 (9)	
H3	0.3041	0.4001	0.0910	0.098*	
C4	0.5603 (6)	0.5189 (3)	0.10952 (17)	0.0851 (9)	
H4	0.5218	0.5875	0.1309	0.102*	
C5	0.7469 (5)	0.5298 (3)	0.10189 (17)	0.0813 (8)	
H5	0.8339	0.6061	0.1181	0.098*	
C6	0.8074 (4)	0.4290 (2)	0.07053 (14)	0.0628 (6)	
H6	0.9341	0.4364	0.0656	0.075*	
C7	0.8515 (3)	0.13091 (19)	0.15128 (11)	0.0434 (5)	
C8	0.8065 (3)	0.04413 (19)	0.21252 (11)	0.0430 (5)	
C9	0.7874 (3)	0.0905 (2)	0.28521 (12)	0.0519 (5)	
H13B	0.8026	0.1775	0.2962	0.062*	0.164 (2)
C10	0.7457 (4)	0.0082 (3)	0.34149 (15)	0.0696 (7)	
H10	0.7321	0.0399	0.3899	0.083*	
C11	0.7248 (4)	−0.1190 (3)	0.32582 (18)	0.0775 (8)	
H11	0.6967	−0.1738	0.3637	0.093*	
C12	0.7444 (4)	−0.1672 (3)	0.25526 (18)	0.0727 (7)	
H12	0.7317	−0.2542	0.2451	0.087*	
C13	0.7833 (3)	−0.0856 (2)	0.19942 (14)	0.0568 (6)	
H13A	0.7944	−0.1190	0.1511	0.068*	0.836 (2)
Cl2	0.23536 (16)	0.10580 (9)	0.45805 (4)	0.1023 (3)	
S2	0.29519 (8)	0.52286 (5)	0.28419 (3)	0.04751 (15)	
O4	0.1951 (3)	0.54542 (16)	0.21405 (9)	0.0656 (5)	
O5	0.4931 (2)	0.57334 (16)	0.30069 (11)	0.0668 (5)	
O6	0.4183 (3)	0.35322 (17)	0.39635 (9)	0.0690 (5)	
N2	0.2562 (2)	0.36633 (16)	0.28095 (10)	0.0435 (4)	
H2N	0.175 (3)	0.327 (2)	0.2468 (11)	0.052*	
C14	0.1875 (3)	0.57205 (18)	0.36012 (12)	0.0446 (5)	
C15	−0.0041 (3)	0.5674 (2)	0.34812 (15)	0.0566 (6)	
H15	−0.0752	0.5370	0.3010	0.068*	
C16	−0.0869 (4)	0.6085 (3)	0.40744 (17)	0.0702 (7)	
H16	−0.2158	0.6045	0.4008	0.084*	
C17	0.0204 (4)	0.6556 (3)	0.47655 (17)	0.0726 (7)	
H17	−0.0365	0.6843	0.5161	0.087*	
C18	0.2094 (4)	0.6607 (3)	0.48769 (15)	0.0662 (7)	
H18	0.2805	0.6930	0.5346	0.079*	
C19	0.2947 (3)	0.6181 (2)	0.42967 (13)	0.0533 (5)	
H19	0.4230	0.6204	0.4372	0.064*	
C20	0.3323 (3)	0.3006 (2)	0.33667 (11)	0.0437 (5)	
C21	0.3018 (3)	0.16165 (19)	0.31408 (12)	0.0429 (4)	
C22	0.2646 (3)	0.0685 (2)	0.36477 (14)	0.0568 (6)	
C23	0.2407 (4)	−0.0596 (3)	0.3413 (2)	0.0743 (8)	
H23	0.2131	−0.1209	0.3757	0.089*	
C24	0.2578 (4)	−0.0950 (3)	0.2678 (2)	0.0776 (8)	
H24	0.2428	−0.1805	0.2523	0.093*	
C25	0.2967 (4)	−0.0054 (3)	0.21684 (17)	0.0689 (7)	
H25	0.3091	−0.0300	0.1669	0.083*	
C26	0.3177 (3)	0.1217 (2)	0.23930 (13)	0.0515 (5)	
H26	0.3429	0.1817	0.2040	0.062*	

### Atomic displacement parameters (Å^2^)

**Table:**

	*U*^11^	*U*^22^	*U*^33^	*U*^12^	*U*^13^	*U*^23^
Cl1A	0.0738 (5)	0.0520 (4)	0.0757 (5)	−0.0070 (3)	0.0281 (4)	−0.0179 (3)
Cl1B	0.091 (3)	0.050 (2)	0.055 (2)	0.0276 (19)	−0.0028 (18)	−0.0126 (15)
S1	0.0569 (3)	0.0613 (4)	0.0326 (3)	−0.0079 (3)	0.0006 (2)	0.0020 (2)
O1	0.0812 (12)	0.0787 (12)	0.0339 (8)	−0.0156 (9)	−0.0091 (8)	0.0000 (8)
O2	0.0661 (11)	0.0814 (13)	0.0610 (10)	−0.0014 (9)	0.0200 (9)	0.0044 (9)
O3	0.0544 (9)	0.0511 (9)	0.0462 (8)	−0.0111 (7)	−0.0141 (7)	0.0070 (7)
N1	0.0528 (11)	0.0503 (11)	0.0386 (9)	−0.0097 (8)	−0.0066 (8)	0.0021 (8)
C1	0.0548 (13)	0.0623 (14)	0.0334 (10)	0.0021 (10)	−0.0014 (9)	0.0125 (9)
C2	0.0586 (15)	0.091 (2)	0.0481 (13)	0.0007 (14)	0.0062 (11)	0.0157 (13)
C3	0.0758 (19)	0.118 (3)	0.0602 (16)	0.0336 (19)	0.0178 (14)	0.0238 (17)
C4	0.110 (3)	0.093 (2)	0.0608 (17)	0.039 (2)	0.0141 (17)	0.0143 (16)
C5	0.102 (2)	0.0620 (17)	0.0726 (18)	0.0102 (16)	−0.0015 (17)	0.0043 (14)
C6	0.0606 (15)	0.0611 (15)	0.0589 (14)	0.0022 (12)	−0.0020 (11)	0.0095 (12)
C7	0.0413 (10)	0.0411 (11)	0.0410 (10)	0.0019 (9)	−0.0047 (8)	−0.0005 (8)
C8	0.0359 (10)	0.0459 (11)	0.0406 (10)	0.0008 (8)	−0.0038 (8)	0.0038 (8)
C9	0.0401 (11)	0.0575 (13)	0.0489 (12)	−0.0036 (9)	0.0002 (9)	−0.0010 (10)
C10	0.0580 (15)	0.095 (2)	0.0468 (13)	−0.0030 (14)	0.0085 (11)	0.0107 (13)
C11	0.0737 (18)	0.081 (2)	0.0735 (19)	0.0039 (15)	0.0069 (14)	0.0341 (16)
C12	0.0743 (17)	0.0556 (15)	0.087 (2)	0.0093 (13)	0.0099 (15)	0.0240 (14)
C13	0.0589 (14)	0.0477 (13)	0.0601 (14)	0.0073 (10)	0.0029 (11)	0.0055 (10)
Cl2	0.1560 (9)	0.1102 (7)	0.0606 (4)	0.0553 (6)	0.0380 (5)	0.0304 (4)
S2	0.0530 (3)	0.0358 (3)	0.0472 (3)	0.0006 (2)	0.0003 (2)	0.0021 (2)
O4	0.0932 (13)	0.0518 (10)	0.0497 (9)	0.0166 (9)	−0.0012 (8)	0.0118 (7)
O5	0.0538 (9)	0.0578 (10)	0.0762 (11)	−0.0117 (8)	0.0120 (8)	−0.0072 (8)
O6	0.0854 (12)	0.0619 (10)	0.0536 (10)	0.0279 (9)	−0.0276 (9)	−0.0126 (8)
N2	0.0445 (9)	0.0359 (9)	0.0430 (9)	0.0046 (7)	−0.0099 (7)	−0.0036 (7)
C14	0.0483 (11)	0.0318 (10)	0.0508 (11)	0.0082 (8)	−0.0018 (9)	0.0021 (8)
C15	0.0538 (13)	0.0473 (12)	0.0654 (14)	0.0139 (10)	−0.0096 (11)	0.0058 (11)
C16	0.0562 (15)	0.0707 (17)	0.091 (2)	0.0311 (13)	0.0045 (14)	0.0136 (15)
C17	0.087 (2)	0.0755 (18)	0.0693 (17)	0.0443 (16)	0.0174 (15)	0.0069 (14)
C18	0.0777 (18)	0.0701 (16)	0.0520 (14)	0.0290 (14)	−0.0031 (12)	−0.0059 (12)
C19	0.0502 (12)	0.0504 (12)	0.0556 (13)	0.0129 (10)	−0.0056 (10)	−0.0037 (10)
C20	0.0420 (11)	0.0476 (11)	0.0415 (11)	0.0163 (9)	−0.0031 (8)	−0.0019 (9)
C21	0.0389 (10)	0.0435 (11)	0.0478 (11)	0.0149 (8)	0.0033 (8)	0.0011 (9)
C22	0.0599 (14)	0.0578 (14)	0.0602 (14)	0.0251 (11)	0.0128 (11)	0.0132 (11)
C23	0.0720 (17)	0.0556 (15)	0.102 (2)	0.0234 (13)	0.0166 (16)	0.0241 (15)
C24	0.0751 (18)	0.0477 (15)	0.111 (2)	0.0223 (13)	0.0070 (17)	−0.0074 (15)
C25	0.0631 (15)	0.0697 (17)	0.0738 (17)	0.0223 (13)	0.0086 (13)	−0.0216 (14)
C26	0.0457 (12)	0.0537 (13)	0.0549 (13)	0.0145 (10)	0.0053 (10)	−0.0055 (10)

### Geometric parameters (Å, °)

**Table:**

Cl1A—C9	1.687 (2)	C13—H13A	0.93
Cl1B—C13	1.565 (4)	Cl2—C22	1.727 (3)
S1—O2	1.4137 (18)	S2—O5	1.4201 (17)
S1—O1	1.4320 (16)	S2—O4	1.4261 (16)
S1—N1	1.6683 (19)	S2—N2	1.6506 (17)
S1—C1	1.744 (3)	S2—C14	1.754 (2)
O3—C7	1.211 (2)	O6—C20	1.204 (2)
N1—C7	1.376 (2)	N2—C20	1.383 (3)
N1—H1N	0.845 (17)	N2—H2N	0.822 (16)
C1—C2	1.389 (3)	C14—C19	1.380 (3)
C1—C6	1.389 (3)	C14—C15	1.386 (3)
C2—C3	1.366 (4)	C15—C16	1.376 (4)
C2—H2	0.93	C15—H15	0.93
C3—C4	1.373 (5)	C16—C17	1.377 (4)
C3—H3	0.93	C16—H16	0.93
C4—C5	1.371 (5)	C17—C18	1.366 (4)
C4—H4	0.93	C17—H17	0.93
C5—C6	1.381 (4)	C18—C19	1.375 (3)
C5—H5	0.93	C18—H18	0.93
C6—H6	0.93	C19—H19	0.93
C7—C8	1.490 (3)	C20—C21	1.495 (3)
C8—C13	1.380 (3)	C21—C22	1.388 (3)
C8—C9	1.389 (3)	C21—C26	1.396 (3)
C9—C10	1.387 (3)	C22—C23	1.392 (4)
C9—H13B	0.93	C23—C24	1.363 (4)
C10—C11	1.361 (4)	C23—H23	0.93
C10—H10	0.93	C24—C25	1.366 (4)
C11—C12	1.363 (4)	C24—H24	0.93
C11—H11	0.93	C25—C26	1.380 (3)
C12—C13	1.373 (3)	C25—H25	0.93
C12—H12	0.93	C26—H26	0.93
			
O2—S1—O1	119.45 (11)	C12—C13—H13A	118.8
O2—S1—N1	109.51 (11)	C8—C13—H13A	118.8
O1—S1—N1	102.84 (10)	O5—S2—O4	119.97 (11)
O2—S1—C1	110.60 (11)	O5—S2—N2	107.85 (10)
O1—S1—C1	109.12 (12)	O4—S2—N2	103.72 (9)
N1—S1—C1	103.99 (10)	O5—S2—C14	108.61 (10)
C7—N1—S1	122.29 (15)	O4—S2—C14	108.75 (11)
C7—N1—H1N	119.6 (17)	N2—S2—C14	107.22 (10)
S1—N1—H1N	115.2 (17)	C20—N2—S2	124.21 (14)
C2—C1—C6	120.7 (3)	C20—N2—H2N	119.4 (17)
C2—C1—S1	119.5 (2)	S2—N2—H2N	115.8 (17)
C6—C1—S1	119.7 (2)	C19—C14—C15	121.1 (2)
C3—C2—C1	119.4 (3)	C19—C14—S2	119.95 (17)
C3—C2—H2	120.3	C15—C14—S2	118.89 (17)
C1—C2—H2	120.3	C16—C15—C14	118.6 (2)
C2—C3—C4	120.5 (3)	C16—C15—H15	120.7
C2—C3—H3	119.7	C14—C15—H15	120.7
C4—C3—H3	119.7	C15—C16—C17	120.2 (2)
C5—C4—C3	120.1 (3)	C15—C16—H16	119.9
C5—C4—H4	120.0	C17—C16—H16	119.9
C3—C4—H4	120.0	C18—C17—C16	120.8 (3)
C4—C5—C6	121.0 (3)	C18—C17—H17	119.6
C4—C5—H5	119.5	C16—C17—H17	119.6
C6—C5—H5	119.5	C17—C18—C19	120.1 (2)
C5—C6—C1	118.3 (3)	C17—C18—H18	120.0
C5—C6—H6	120.8	C19—C18—H18	120.0
C1—C6—H6	120.8	C18—C19—C14	119.2 (2)
O3—C7—N1	120.84 (19)	C18—C19—H19	120.4
O3—C7—C8	124.36 (17)	C14—C19—H19	120.4
N1—C7—C8	114.79 (17)	O6—C20—N2	121.96 (19)
C13—C8—C9	117.3 (2)	O6—C20—C21	124.54 (19)
C13—C8—C7	121.28 (19)	N2—C20—C21	113.47 (16)
C9—C8—C7	121.41 (19)	C22—C21—C26	117.3 (2)
C10—C9—C8	120.6 (2)	C22—C21—C20	123.27 (19)
C10—C9—Cl1A	117.6 (2)	C26—C21—C20	119.43 (19)
C8—C9—Cl1A	121.79 (18)	C21—C22—C23	121.1 (2)
C10—C9—H13B	119.7	C21—C22—Cl2	121.50 (18)
C8—C9—H13B	119.7	C23—C22—Cl2	117.3 (2)
C11—C10—C9	119.9 (3)	C24—C23—C22	119.9 (3)
C11—C10—H10	120.0	C24—C23—H23	120.0
C9—C10—H10	120.0	C22—C23—H23	120.0
C10—C11—C12	120.9 (3)	C23—C24—C25	120.3 (3)
C10—C11—H11	119.6	C23—C24—H24	119.9
C12—C11—H11	119.6	C25—C24—H24	119.9
C11—C12—C13	119.0 (3)	C24—C25—C26	120.2 (3)
C11—C12—H12	120.5	C24—C25—H25	119.9
C13—C12—H12	120.5	C26—C25—H25	119.9
C12—C13—C8	122.3 (2)	C25—C26—C21	121.2 (2)
C12—C13—Cl1B	115.1 (3)	C25—C26—H26	119.4
C8—C13—Cl1B	121.4 (2)	C21—C26—H26	119.4
			
O2—S1—N1—C7	59.3 (2)	C7—C8—C13—Cl1B	11.9 (3)
O1—S1—N1—C7	−172.73 (19)	O5—S2—N2—C20	49.5 (2)
C1—S1—N1—C7	−59.0 (2)	O4—S2—N2—C20	177.73 (18)
O2—S1—C1—C2	166.94 (17)	C14—S2—N2—C20	−67.30 (19)
O1—S1—C1—C2	33.6 (2)	O5—S2—C14—C19	−16.8 (2)
N1—S1—C1—C2	−75.60 (19)	O4—S2—C14—C19	−148.99 (18)
O2—S1—C1—C6	−15.2 (2)	N2—S2—C14—C19	99.45 (18)
O1—S1—C1—C6	−148.54 (18)	O5—S2—C14—C15	161.18 (17)
N1—S1—C1—C6	102.27 (19)	O4—S2—C14—C15	29.0 (2)
C6—C1—C2—C3	−0.4 (3)	N2—S2—C14—C15	−82.52 (18)
S1—C1—C2—C3	177.40 (19)	C19—C14—C15—C16	−0.7 (3)
C1—C2—C3—C4	0.5 (4)	S2—C14—C15—C16	−178.68 (19)
C2—C3—C4—C5	−0.2 (4)	C14—C15—C16—C17	1.3 (4)
C3—C4—C5—C6	−0.3 (5)	C15—C16—C17—C18	−0.9 (4)
C4—C5—C6—C1	0.3 (4)	C16—C17—C18—C19	−0.2 (4)
C2—C1—C6—C5	0.0 (3)	C17—C18—C19—C14	0.8 (4)
S1—C1—C6—C5	−177.8 (2)	C15—C14—C19—C18	−0.4 (3)
S1—N1—C7—O3	−10.0 (3)	S2—C14—C19—C18	177.59 (19)
S1—N1—C7—C8	169.48 (16)	S2—N2—C20—O6	7.9 (3)
O3—C7—C8—C13	−134.5 (2)	S2—N2—C20—C21	−170.30 (15)
N1—C7—C8—C13	46.0 (3)	O6—C20—C21—C22	39.0 (3)
O3—C7—C8—C9	45.0 (3)	N2—C20—C21—C22	−142.8 (2)
N1—C7—C8—C9	−134.4 (2)	O6—C20—C21—C26	−138.5 (2)
C13—C8—C9—C10	−0.3 (3)	N2—C20—C21—C26	39.6 (3)
C7—C8—C9—C10	−179.9 (2)	C26—C21—C22—C23	−1.2 (3)
C13—C8—C9—Cl1A	−178.19 (16)	C20—C21—C22—C23	−178.8 (2)
C7—C8—C9—Cl1A	2.2 (3)	C26—C21—C22—Cl2	−178.16 (17)
C8—C9—C10—C11	0.5 (4)	C20—C21—C22—Cl2	4.3 (3)
Cl1A—C9—C10—C11	178.5 (2)	C21—C22—C23—C24	1.4 (4)
C9—C10—C11—C12	0.1 (4)	Cl2—C22—C23—C24	178.5 (2)
C10—C11—C12—C13	−0.9 (4)	C22—C23—C24—C25	−0.5 (4)
C11—C12—C13—C8	1.1 (4)	C23—C24—C25—C26	−0.5 (4)
C11—C12—C13—Cl1B	169.1 (3)	C24—C25—C26—C21	0.7 (4)
C9—C8—C13—C12	−0.5 (3)	C22—C21—C26—C25	0.2 (3)
C7—C8—C13—C12	179.1 (2)	C20—C21—C26—C25	177.9 (2)
C9—C8—C13—Cl1B	−167.7 (2)		

### Hydrogen-bond geometry (Å, °)

**Table:**

*D*—H···*A*	*D*—H	H···*A*	*D*···*A*	*D*—H···*A*
N1—H1N···O1^i^	0.84 (2)	2.09 (2)	2.922 (2)	168 (2)
N2—H2N···O3^ii^	0.82 (2)	2.06 (2)	2.883 (2)	179 (3)

Symmetry codes: (i) −*x*+1, −*y*, −*z*; (ii) *x*−1, *y*, *z*.

## References

[bb1] Gowda, B. T., Foro, S., Suchetan, P. A. & Fuess, H. (2009*a*). *Acta Cryst.* E**65**, o2516. 10.1107/S1600536809037222 PMC2970249 21577963

[bb2] Gowda, B. T., Foro, S., Suchetan, P. A. & Fuess, H. (2009*b*). *Acta Cryst.* E**65**, o2750. 10.1107/S1600536809041051 PMC2971321 21578344

[bb3] Oxford Diffraction (2009). *CrysAlis CCD* and *CrysAlis RED* Oxford Diffraction Ltd, Yarnton, England.

[bb4] Sheldrick, G. M. (2008). *Acta Cryst.* A**64**, 112–122. 10.1107/S0108767307043930 18156677

[bb5] Spek, A. L. (2009). *Acta Cryst.* D**65**, 148–155. 10.1107/S090744490804362X PMC2631630 19171970

[bb6] Suchetan, P. A., Gowda, B. T., Foro, S. & Fuess, H. (2009). *Acta Cryst.* E**65**, o3156. 10.1107/S1600536809048399 PMC2971909 21578874

